# Pancytopenia, Recurrent Infection, Poor Wound Healing, Heterotopia of the Brain Probably Associated with A Candidate Novel de Novo *CDC42* Gene Defect: Expanding the Molecular and Phenotypic Spectrum

**DOI:** 10.3390/genes12020294

**Published:** 2021-02-20

**Authors:** Abdulaziz Asiri, Deemah Alwadaani, Muhammad Umair, Kheloud M. Alhamoudi, Mohammed H. Almuhanna, Abdul Nasir, Bahauddeen M. Alrfaei, Abeer Al Tuwaijri, Tlili Barhoumi, Yusra Alyafee, Bader Almuzzaini, Mohammed Aldrees, Mariam Ballow, Latifah Alayyar, Abdulkareem Al Abdulrahman, Yazeid Alhaidan, Nahlah Al Ghasham, Sulaiman Al-Ajaji, Mohammad Alsalamah, Wafa Al Suwairi, Majid Alfadhel

**Affiliations:** 1 Faculty of Applied Medical Sciences, University of Bisha, 255, Al Nakhil, Bisha 67714, Saudi Arabia; dr.abdulaziz.asiri@gmail.com; 2 Medical Genomics Research Department, King Abdullah International Medical Research Center (KAIMRC), King Saud Bin Abdulaziz University for Health Sciences, King AbdulAziz Medical City, Ministry of National Guard Health Affairs (MNG-HA), Riyadh 11426, Saudi Arabia; alwadaanide@NGHA.MED.SA (D.A.); Umairmu@ngha.med.sa (M.U.); AlHamoudiKh@NGHA.MED.SA (K.M.A.); altuwaijriab@ngha.med.sa (A.A.T.); ahmedyu@ngha.med.sa (Y.A.); MuzainiB@NGHA.MED.SA (B.A.); aldressmo@NGHA.MED.SA (M.A.); BallowMa@NGHA.MED.SA (M.B.); alayyarla@NGHA.MED.SA (L.A.); alabdulrahmanab@NGHA.MED.SA (A.A.A.); alhaidanya@NGHA.MED.SA (Y.A.); 3 Cellular Therapy and Cancer Research Department, King Abdullah International Medical Research Center, King Saud Bin Abdulaziz University for Health Sciences, King Abdulaziz Medical City, Ministry of National Guard Health Affairs (MNG-HA), Riyadh 11426, Saudi Arabia; moha.almuhanna@gmail.com; 4 Department of Molecular Science and Technology, Ajou University, Suwon 443-749, Korea; nasirkhan@ajou.ac.kr; 5 Stem Cells Department, King Abdullah International Medical Research Center, King Saud Bin Abdulaziz University for Health Sciences, King Abdulaziz Medical City, Ministry of National Guard Health Affairs (MNG-HA), Riyadh 11426, Saudi Arabia; alrfaeiba@ngha.med.sa; 6 Medical Core Facility and Research Platforms, King Abdullah International Research Center (KAIMRC), King Saud Bin Abdulaziz University for Health Sciences, King Abdulaziz Medical City, Ministry of National Guard Health Affairs, Riyadh 11426, Saudi Arabia; barhoumitl@ngha.med.sa; 7 Hematology Division, Department of Pathology and Laboratory Medicine, King Abdulaziz Medical City, Riyadh 11426, Saudi Arabia; Alghashamna@NGHA.MED.SA; 8 Allergy and Immunology Division, Department of Pediatrics, King Abdullah Specialist Children’s Hospital, King Saud Bin Abdulaziz University for Health Sciences, King Abdulaziz Medical City, Ministry of National Guard Health Affairs (MNG-HA), Riyadh 11426, Saudi Arabia; Ajaji@NGHA.MED.SA (S.A.-A.); alsalamahmo@NGHA.MED.SA (M.A.); 9 Rheumatology Division, Department of Pediatrics, King Abdullah Specialist Children’s Hospital, King Saud Bin Abdulaziz University for Health Sciences, King Abdulaziz Medical City, Ministry of National Guard Health Affairs (MNG-HA), Riyadh 11426, Saudi Arabia; SewairiW@NGHA.MED.SA; 10 Genetics and Precision Medicine Department (GPM), King Abdullah Specialized Children’s Hospital, King Saud Bin Abdulaziz University for Health Sciences, King Abdulaziz Medical City, Ministry of National Guard Health Affairs (MNG-HA), Riyadh 11426, Saudi Arabia

**Keywords:** *CDC42*, a de novo missense variant, poor wound healing, pancytopenia, recurrent infections

## Abstract

CDC42 (cell division cycle protein 42) belongs to the Rho GTPase family that is known to control the signaling axis that regulates several cellular functions, including cell cycle progression, migration, and proliferation. However, the functional characterization of the *CDC42* gene in mammalian physiology remains largely unclear. Here, we report the genetic and functional characterization of a non-consanguineous Saudi family with a single affected individual. Clinical examinations revealed poor wound healing, heterotopia of the brain, pancytopenia, and recurrent infections. Whole exome sequencing revealed a de novo missense variant (c.101C > A, p.Pro34Gln) in the *CDC42* gene. The functional assays revealed a substantial reduction in the growth and motility of the patient cells as compared to the normal cells control. Homology three-dimensional (3-D) modeling of CDC42 revealed that the Pro34 is important for the proper protein secondary structure. In conclusion, we report a candidate disease-causing variant, which requires further confirmation for the etiology of *CDC42* pathogenesis. This represents the first case from the Saudi population. The current study adds to the spectrum of mutations in the *CDC42* gene that might help in genetic counseling and contributes to the CDC42-related genetic and functional characterization. However, further studies into the molecular mechanisms that are involved are needed in order to determine the role of the *CDC42* gene associated with aberrant cell migration and immune response.

## 1. Background

Skin is the most important protector against several harmful external factors, including bacteria and viruses; thus, a complete and efficient repair of the skin wound is critical. Skin wound repair is a normal complex process, which requires the stimulation or recruitment of various cell types, including endothelial cells, fibroblast, keratinocytes, and inflammatory cells [1,2]. Several growth factors and cytokines that involve transforming growth factor (TGF)–β, interleukin–1 (IL–1)–β, epidermal growth factor (EGF), fibroblast growth factor (FGF), tumor necrosis factor (TNF)–α, and platelet–derived growth factor (PDGF) have shown to play vital roles in driving these processes forward in a harmonized manner [3,4].

CDC42 (also known as cell division cycle protein) is a member of the Rho GTPase family that is known to regulate signaling pathways that control several cellular functions, including cell cycle progression, migration, proliferation, cell morphology, and endocytosis [5]. Like other Rho family members, CDC42 cycles between the active, GTP- bound state, and the inactive, GDP-bound state in the cells, and it is tightly regulated by various signaling pathways under physiologic conditions [6,7]. Previous studies have reported that de novo missense variants in the *CDC42* gene can often result in a number of clinical manifestations, including developmental delay, facial dysmorphism, recurrent infections, and thrombocytopenia [8,9,10,11]. Another study reported that the dysfunction of CDC42 may delay skin wound healing processes by increasing the expression of IL–1β and TNF–α in endothelial cells [12]. A recent report also indicates a prominent role for CDC42 in the regulation of cell polarity and growth [6]. However, the mechanisms that underlie the *CDC42* gene mutation resulting in variable clinical phenotypes remain to be elucidated. 

Here, we report a 19-year-old Saudi descendant female presenting with chronic pancytopenia, recurrent infections, poor wound healing, and with an MRI brain showed migration anomaly in the form of sub ependymal heterotopia and multiple heterotopic islands in the right frontal white matter. Using WES, we identified a novel de novo variant (c.101C > A:p.P34Q) in the *CDC42* gene, which was not detected in her parents and two healthy siblings, which will add to the molecular and phenotypic profile of this syndrome.

## 2. Materials and Methods

### 2.1. Human Subjects

The proband underwent a full routine clinical evaluation, including history examination, hematological and immunological investigations, radiological, and several rheumatology and genetics evaluations were conducted at King Abdulaziz Medical City in Riyadh, Saudi Arabia. Specimen collection was obtained by a clinical geneticist and sent for WES and other genetic tests to assess the multisystem disorder.

### 2.2. Ethical Approval

All of the family members provided written informed consent to participate in this study. The Institution Review Board of KAIMRC approved the study protocols, study number: RC19/120/R. The study was conducted under the tenets of the Declaration of Helsinki. Written informed consent was obtained from the patient’s parents for the publication of images.

### 2.3. DNA Extraction

The blood samples were taken from all family members and DNA was then extracted following the standard protocols using QIAamp Blood Midi Kit. Next, the extracted DNA quantity and purity were determined using a Nanodrop-1000 spectrophotometer.

### 2.4. Whole Exome Sequencing (WES)

WES was performed on the genomic DNA of the affected individual and other family members using the Illumina HiSeq 2500 platform to capture regions of interest from the fragmented DNA library (MDL, KFSH & RC, Riyadh, Saudi Arabia). A minimum coverage of 30× of 95% of the target regions was performed, respectively. The sequence data from the affected individual were compared and mapped to the human genome build UCSC hg19 reference sequence. The quality and coverage assessment for targeted coding exons of the protein-coding genes was performed. Variants that were filtered after WES were characterized using the American College of Medical Genetics and Genomics (ACMG) guidelines.

### 2.5. Bioinformatics Analysis 

The potential effect of the identified variant was predicted using four different prediction tools including, MutationTaster, Mutation Assessor, Sorting Intolerant From Tolerant (SIFT), and PROVEAN. The identified variant was searched in different public databases, including Exome Aggregation Consortium (ExAC), Genome Aggregation Database (gnomAD), Exome Variant Server (EVS), 1000 Genomes, and Single Nucleotide Polymorphism Database (dbSNP).

### 2.6. Mutation Confirmation and Sequencing Analysis

Sanger sequencing was carried out in order to confirm the segregation of the identified variant in all family members. The identified missense mutation in *CDC42* gene (NM_001791.4: c.101C > A) was validated using primers: F: 5′-AGTGTGTTGTTGTGGGCGAT-3′ and R: 5′- TGTCACCCCTTCTGACTTTCC -3′.

### 2.7. Cell Isolation and Culture

A skin biopsy was taken from the patient and fibroblast was then separately isolated using the explant method, as previously described [13]. The normal cell control is a normal foreskin fibroblast purchased from ATCC (Hs27; CRL-1634) and it was isolated from newborn black male. The cells were cultured in Dulbecco’s Modified Eagles Medium (DMEM) supplemented with 10% fetal bovine serum (FBS), 10 U/mL penicillin/Streptomycin, and 2 mM L-glutamine and maintained at 37 °C in a 5% CO2 atmosphere. Validation was performed, as previously described [13].

### 2.8. Cell Treatment 

Cells were treated using EGF and FGF Recombinant Human Protein in order to stimulate CDC42 signaling pathways (Thermo Scientific, Waltham, MA, USA). Briefly, the cells were seeded in a six-well plate and starved in a serum-free DMEM for 24 h before stimulation. The cells were then treated with 20 ng/mL EGF or FGF in a total volume of 2 mL per well of the growth DMEM media (supplemented with 10% FBS). 

### 2.9. Wound Healing Assay

The wound healing assay was applied to assess cell migration in the patient. In brief, 5 × 10^5^ were seeded into culture-insert 2 well (Ibidi) that was attached in a six-well plate and incubated at 37 °C for 24 h. Following this, the wound was generated by removing the inserts, and photos were captured at different time points at 10× magnification using the EVOS FL Auto Imaging System (Life Technologies, Bothell, WA, USA). The free gap width was approximately 500 microns (±50 microns) at 0 h. Cell migration was quantified by measuring the remaining area of the wound by ImageJ software.

### 2.10. Cell Viability Assay

CellTiter-Glo^®^ Luminescent Cell Viability Assay (Promega, Madison, WI, USA) was used as an indirect method for determining the number of live cells in culture based on the quantification of the ATP present. In brief, 5000 cells were seeded into a 96 well plate and then incubated for 24 h. Next, 100 μL of CellTiter-Glo^®^ Reagent was directly applied to the cells and without cells as control and then incubated for 1 h at 37 °C. Following this, the relative luminescent units of each well were then measured using EnVision^®^ Multimode Plate Reader. The blank luminescence reading was subtracted from each experimental luminescence, and the blank corrected values were then normalized to the normal cells control. 

### 2.11. Cell Cycle Assay 

Cell cycle was assessed by propidium iodide (PI) staining (Life Technologies) and fluorescence-activated cell sorting (BD FACS Canto II flow cytometer, BD Biosciences). The cells were harvested and collected by centrifugation at 200 g for 3 min. The cells were fixed using ice-cold 70% ethanol in 1X PBS (Gibco) and then stored at −20 °C overnight. After incubation, the cells were washed, centrifuged, treated with 10 μg/mL RNase A (Invitrogen), and then re-suspended in PBS containing 50 μg/mL PI buffer solution. The negative control cells were prepared without staining and data were acquired for unstained cells. Independent control cells Hs27 (ATCC: CRL-1634) were used as a standard control. At least twenty thousand cells were analyzed in one parameter mode and the calculations were carried out using the FACSDiva software Version 6.1.3 (BD Biosciences) for cell cycle analysis.

### 2.12. Protein Modeling

It has been documented that the guanine nucleotide dissociation inhibitor (GDI), which is a family of small GTPases, acts as a significant signaling inhibitor via Rho family GTP-binding proteins (CDC42). The crystal structure of CDC42 with RhoGDI-1 was retrieved from the Protein Data Bank (PDB) with accession number 1DOA [14]. The mutant structure was modeled and energy minimized using Discovery Studio 2.0 (Accelrys) in order to elucidate the impact of substituted proline to glutamine at residue 34. The electrostatic potential surface of wild and mutant CDC42 was calculated using the PyMOL-based “apbsplugin.py” tool. Figures were made using the PyMOL molecular viewer https://pymol.org/ (accessed on 16 January 2021).

### 2.13. Statistical Analysis

GraphPad Prism (version 8.1) software was used for the statistical analysis. The results were analyzed by either unpaired T-test or analysis of variance (two-way *ANOVA*), and *p* < 0.05 was considered to be significant.

## 3. Results

### 3.1. Clinical Description

**Figure 1:**
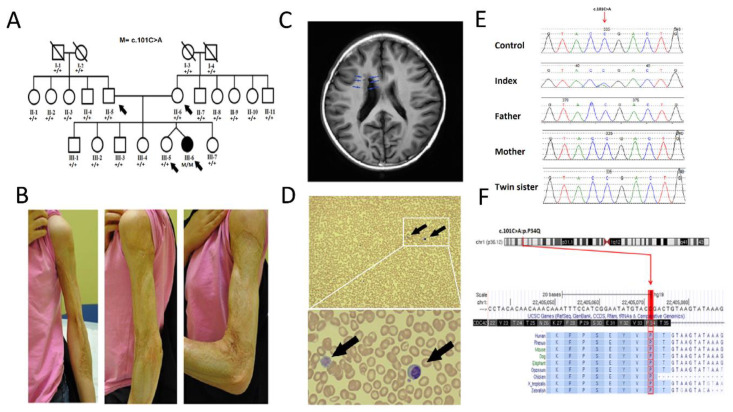
Identification of a novel de novo missense variant (c.101C > A: p.P34Q) in CDC42 in a single patient with poor wound healing and recurrent infection. (**A**) Pedigree of the analyzed family members. The allelic status is given below each individuals. Symbols as follows: empty, unaffected; filled, affected; black arrow, individuals subjected to whole exome sequencing (WES). (**B**) Photographs showing the clinical features of the patient including poor wound healing and recurrent infection. Written informed consent was obtained from the patient’s parents for the publication of images. (**C**) Magnetic resonance imaging (MRI) of brain showed migration anomaly in the form of sub ependymal heterotopia and multiple heterotopic islands in the right frontal white matter. (**D**) Peripheral blood smear (×20 power magnification on the upper panel and 50× magnification on the lower panel, May–Grunwald Giemsa stain) showing leukopenia and thrombocytopenia. Only one lymphocyte is available in the field. The black arrow are for the giant platelets. (**E**) Segregation of the identified pathogenic missense variant (c.101C > A) in all family members. (**F**) The alignment of CDC42 from different eukaryotic species shows that the p.P34Q residue is highly conserved.

The proband (III-6) is part of the dizygotic twin and the other twin (III-5) is a healthy sister with no complaint (Figure 1A). She is a product of full term normal spontaneous vaginal delivery with an appropriate Apgar score and growth parameters. The first concern was noted in the first year of life when the parents noticed recurrent upper respiratory tract infections that required several courses of antibiotics. As she grew up, these URTI-like episodes became associated with an intense systemic response with high-grade fever, elevated inflammatory markers, and chronic anemia. She had frequent hospitalization for IV antibiotics. 

Adenotonsillectomy was done at the age of five years, followed by two weeks admission to manage her poor heeling and local inflammation, and then discharged. The patient was then admitted again with severe abdominal pain that mimics appendicitis that requires emergency exploratory laparotomy and it was found to have intestinal gangrene that requires resection of 20 cm of her intestine. At sic years of age, after getting her preschool vaccines (DTaP, MMR), she developed a severe ulcer and gangrene that progressed over six months and required grafting by plastic surgery and treatment with prednisone. At the age of eight years, she had severe left otitis externa and left chronic sterile mastoiditis that necessitates tympanoplasty and mastoidectomy. After that, she had recurrent skin ulcerative lesions that were associated with systemic inflammation with fever and very high inflammatory markers and only responded to high doses of systemic steroids. All of the lesion cultures were negative for any pathogen. At the peak of her illness on repeated occasions, she develops marked non hemolytic anemia, reticulocytopenia, low platelets, and lymphopenia (Table 1).

The chronic use of multiple immunosuppressive medications failed to prevent the recurrence of these episodes. However, a significant spontaneous improvement was noted after puberty. Developmentally, she is appropriate for her age, with no behavioral, autistic, or attention-deficit features. She is in university in designing college with no learning difficulties. She has no skeletal, renal, or cardiovascular abnormalities. 

On examination, the patient’s growth parameters were, as follows: height: 154 cm (5th–10th percentile), weight: 49 kg (10th–25th percentile), and head circumference: 54 cm (25th–50th percentile). She has a big scar that involves the whole left arm from the previous complication after vaccination (Figure 1B). Additionally, she has multiple scars in the abdomen and back due to previous recurrent infections. Magnetic resonance imaging (MRI) of the brain showed migration anomaly in the form of sub ependymal heterotopia and multiple heterotopic islands in the right frontal white matter (Figure 1C). However, this did not affect the patient’s brain function or cause any neurological deficit. Electrocardiogram (ECG) and Echocardiogram were normal, and the skeletal survey reported unremarkable findings.

Laboratory investigations showed white blood cells (WBC): 1.9–3.9 (normal range: 4.00–11.00 × 10^9^/L); haemoglobin (Hb): 84–100 (normal range: 120–160 gm/L) and platelets: 36–124^9^ (normal range: 150–400 × 10^9^/L); erythrocyte sedimentation rate (ESR): 22–120 (normal range: 0–20 mm/hr);C reactive protein: 2–333 (normal range: ≤ 8 mg/L); Prothrombin time (PT):8.9–13.2 (normal range: 9.38–12.34 s); partial thromboplastin time (PTT): 27.3–34.1 (normal range: 24.84–32.96 s); and, international normalized ratio (INR): 0.9–1.24 (normal range: 0.80–1.20). 

Other laboratory investigations were unremarkable, including renal profile; the liver function tests were unremarkable. Initially, the patient had normal neutrophil counts, but, by eight years of age, it started declining with time. Bone marrow aspirate revealed normal myelopoiesis. Via flowcytometric measurements and upon Phorbol Myristate Acetate (PMA) stimulation, the neutrophils had an appropriate expression of CD11b and CD18, as well as robust oxidative burst activity when compared to healthy controls (92% vs. 99% in healthy controls). The T-helper counts were reduced on all occasions (296 cells/microL normal range 500–1400 cells/microL). The B-cells also showed persistently low counts (129 cells/microL [normal range 300–500 cells/microL]). 

However, quantitative immunoglobulins were normal. The patient had protective antibody titers to mumps and rubella, but not to measles. Peripheral blood smear showed giant platelets, leukopenia, and thrombocytopenia (Figure 1D).

### 3.2. Genetic Analysis

Using WES, a de novo missense mutation in *CDC42* (Chr1: 22405072, NM_001791.4, c.101C > A, p.Pro34Gln) affecting both isoforms—specifically exon 2—was identified in the patient (III-6). De novo missense variants of CDC42 are frequently the cause of Takenouchi–Kosaki syndrome (OMIM; 616737). This variant is classified as variant of uncertain significance according to ACMG classification; however, it is of note that this variant has not been reported previously; it was not found in the ExAC/gnomAD or an in-house database of 2000 exomes from unrelated Saudi Arabian individuals. The identified missense variant was predicted pathogenic while using several online in silico tools including Mutation Taster (Disease-causing), Mutation assessor (High), SIFT (Damaging), and PROVEAN (Damaging). Sanger sequencing confirmed the presence of the variant in the proband (III-6) and the absence in the other family member samples, indicating a de novo origin for this variant (Figure 1E). This mutation was located in an evolutionarily conserved N-terminal region of CDC42 (Figure 1F). 

### 3.3. Functional Studies

#### 3.3.1. Mutated CDC42 Selectively Abrogates EGF/FGF Induced Cell Migration and Proliferation

**Figure 2:**
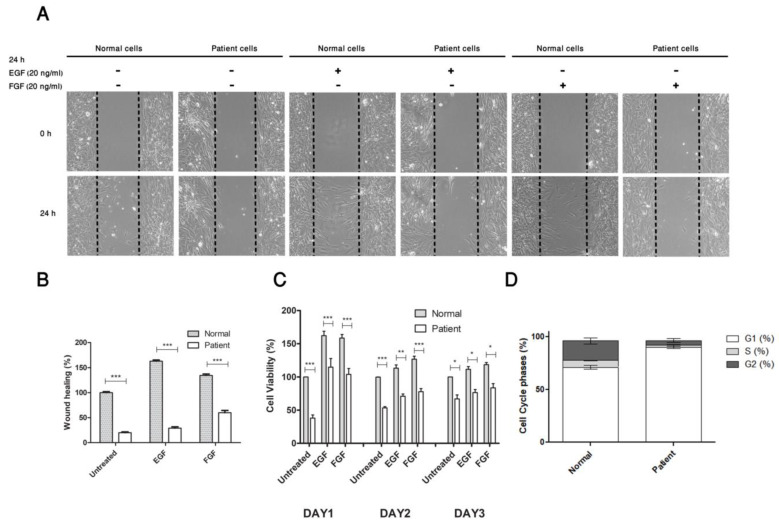
The effect of the potential candidate p.P34Q mutation on cell migration, cell proliferation and cell cycle progression. (**A**,**B**) Wound healing assay showed that stimulation cells with either epidermal growth factor (EGF) or fibroblast growth factor (FGF) treatments (20 ng/mL for 24 h) significantly increases wound closure in normal cells compared to patient cells (*n* = 3, *, *p* < 0.05, **, *p* < 0.01, ***, *p* < 0.001, two-way *ANOVA*). (**C**) Cell viability assay showed that stimulation of EGF/FGF (20 ng/mL for 24 h) did not cause a significant induces in cell proliferation in the patient compared to the normal cells (*n* = 3, *, *p* < 0.05, **, *p* < 0.01, ***, *p* < 0.001, two-way *ANOVA*). (**D**) The distribution of cell cycle phases showed that there were significant increases in G1 phase (*p* = 0.0006), S phase (*p* = 0.0036), and G2 phase (*p* = 0.0134) of patient cells compared to normal control (*n* = 3, two-tailed unpaired t test).

CDC42 is a master regulator of cell migration, proliferation, and cell growth. An in vitro wound healing assay assessed the effect of the identified variant in the *CDC42* gene on polarized migration. Because the patient has poor wound healing, we aimed to investigate whether the identified variant in *CDC42* is responsible for this phenotype using different growth factor stimulators, FGF and EGF. First, we compared the cell migration in normal to the patient cells under untreated conditions. The control cells showed increased wound closure ability as compared to patient cells. (See Supplementary Materials) Interestingly, the cells treated with EGF or FGF exhibited enhanced cell migration in the normal control but failed to promote the patient cells, implying that the identified variant could be responsible for the loss of function of this CDC42 mediated cell growth and migration processes (Figure 2A,B). In addition to the wound healing assay, we investigated whether the identified mutation of *CDC42* affects the growth of the patient cells that were treated with or without growth factor, FGF/EFG stimulators. The cells were stimulated with 20 ng/mL EGF or FGF for a period of 24, 48, and 72 h. Cell viability assays showed that either FGF or EGF treatments did not significantly increase cell proliferation in the patient’s cells in comparison with normal cells control (Figure 2C). Collectively, these findings suggest that de novo missense mutation in *CDC42* may delay skin wound healing processes of the patient by abolishing EGF/FGF induced cell migration and proliferation. 

#### 3.3.2. Substitution of Pro to Gln at Position 34 Induces Arrest in G1 Phase of Patient’s Cell Cycle Progression

Flow cytometric analysis was performed in order to evaluate whether the identified p.P34Q mutation (Pro to Gln at position 34) causes an alteration in cell cycle profile in patients’ cells. Figure 2D showed the quantification of cell cycle phase distribution for the patient’s fibroblast cells compared to the normal control cells. Overall, there was a significant difference in cell cycle phases when compared to the normal cells. There was an increase in the percentage of cells in the G0/G1 cells in the patient’s cells (89.8%) as compared to the control cells (71.2%). Similarly, the percentage of the patient cells in the S- phase and G2 phase was (1.83%) and (4.71%), respectively, whilst it was (6.44%) and (18.34%) in the normal control. The reduction in the level of the cells entering S- and G2- phase is likely due to the significant delay in G0/G1- phase progression and suggest that the reduction in cell cycle progression is probably induced by the p. P34Q mutation in the *CDC42* gene and it contributed to the overall reduction of cellular development. This is also supported by the finding of the reduced proliferation of the patient’s fibroblast (Figure 2C). This suggests that the dysfunction of CDC42 due to the identified mutation (p.P34Q) could be responsible for the reduction in cell cycle progression in the patients cells. 

#### 3.3.3. p.P34Q Mutation Affects the Binding of CDC42 to RhoGDI-1 Protein

**Figure 3:**
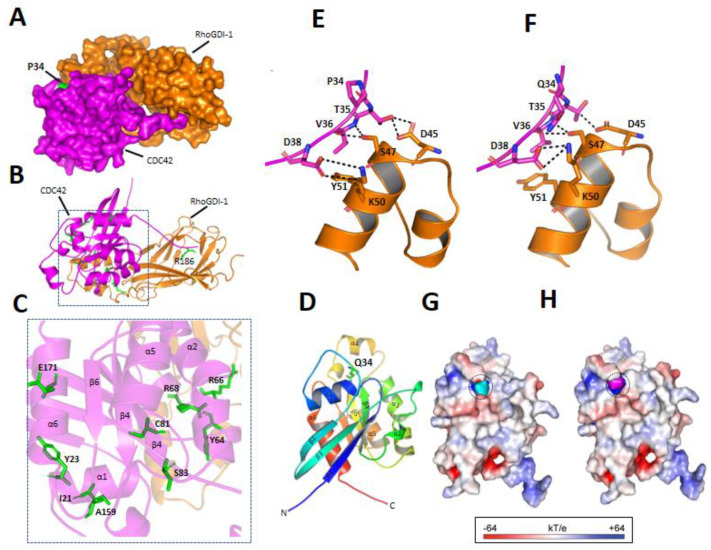
Structural analysis of CDC40-RhoGDI complex for p.P34Q effect. (**A**) Surface and ribbon view (**B**) of Complex structure of CDC40 with RhoGDI. (**C**) Close view of mutant report in CDC40 protein. (**D**) The three-dimensional structure of CDC40. Each α-helices and β-sheet is indicated by a different color. (**E**) The interface interaction at position 34 of wild and mutant (**F**) type CDC40-RhoGDI complex. (**G**) Electrostatic surface view of the wild and mutant type CD40 (**H**) protein. Electrostatic potential is expressed as a spectrum ranging from -64 kT/e (red) to +64 kT/e (blue). Cyan and magenta color indicates P34 and Q34 residue, respectively.

The binding interface of wild and mutant CDC42 with RhoGDI-1 complex was analyzed in order to explore the effect of the identified missense mutation (p.P34Q) on the CDC42 protein. A close review of the binding interface of the wild type and mutant protein revealed different potential interaction patterns. In the case of wild type, Thr35, Val36, and Asp38 of CDC42 display hydrogen bonds with Asp45, Ser47, and Lys50 of RhoGDI-1, respectively. All of these residues, including Pro34, reside in the loop region between the α1 and β2 of CDC42 (Figure 3A–E). The substitution of Pro to Gln at position 34 established new hydrophobic interaction between Val36 and Tyr51 (Figure 3F,G). Moreover, Thr35 in the mutant CDC42 established an ionic and hydrogen interaction with Asp45. The different interaction patterns that are displayed by the wild and mutant protein contribute to the change in nearby surface electrostatic potential (Figure 3H,I). This might lead to unstable interaction between the CDC42 and RhoGDI-1 complex.

## 4. Discussion

Here, we characterize a previously unidentified and distinctive poor wound healing/recurrent infection disorder that is caused by a disease causing candidate of a novel missense variant in the *CDC42* gene (c.101C > A:p.P34Q) by using several functional assays. We provide evidence that the identified variant might cause the disease in the patient and it has unique consequences on the function of *CDC42*, resulting in a perturbation of inflammatory response, immune function, cell migration, proliferation, and wound healing processes.

CDC42 codes for a small GTPase of the Rho family and, through interacting proteins called effectors, controls multiple signaling pathways that regulate several cellular functions, including cell polarity and migration, cell cycle progression, and endocytosis [5]. CDC42 consists of five (G1–G5) highly conserved motifs, which functions in phosphate binding (G1 and G3), GTP hydrolysis and binding (G4 and G5), and effector binding (G2) [11,17,18]. More recently, several missense mutations in this gene were found to be linked with developmental anomalies, including intellectual disability, brain malformation, defective growth, and facial dysmorphism, immune, and lymphatic defects [8,11]. Additionally, it has been reported that patients sharing a single de novo missense variant (p.R186C) have been associated with multisystem inflammatory disease, including pancytopenia, skin rash, fever, and hepatosplenomegaly [15]. CDC42 was found to play a critical role in HSCs and other hematopoietic progenitors in mutant mice [19,20]. *CDC42* deletion in the mouse bone marrow led to a rapidly fatal myeloproliferative disorder [20]. Another recent report suggests that mutation in the *CDC42* gene in a human patient is found to be associated with myelofibrosis in adulthood [21]. In the proband studied here, in which a novel, de novo missense p.P34Q mutation in the *CDC42* gene was identified for the first time, being characterized by poor wound healing, pancytopenia, recurrent infections, and thrombocytopenia. However, the identified mutation appeared to be potentially relevant to immunodeficiency and migration anomalies, although the clinical phenotypes described in this study are distinct from what has previously been reported in animal models and other human patients.

The identified p.P34Q mutation in the CDC42 gene was previously reported in a young male patient presenting with an acute myeloid leukaemia (COSMIC database, sample ID: COSS2810409). However, this patient was also found to have a G12D mutation in the NRAS gene. This variant is most probably causing disease in the patient, as the NRAS gene (OMIM: 164790) is already reported to be involved as a cause for an acute myeloid leu-kaemia [22]. Additionally, the current proband and previously reported patients with similar presentations of AML were not part of the phenotype [11]. Therefore, further analysis regarding the role of CDC42 gene in AML patients should be performed.

Cell migration is critical not only during normal development, but throughout life, for several biological processes, including wound repair, angiogenesis, and inflammatory response [23]. The Rho family of small GTPases particularly Rac, Rho, and CDC42 regulate a wide variety of cytoskeletal functions that occur during cell migration [24,25]. Cell motility, which is driven by cytokines and growth factors released concordantly into the injury site, is important during early wound repair. Among growth factors or extracellular signals that directly influence the cells to migrate, EGF and FGF induce migration [26,27]. The activation of CDC42 by FGF stimulation is crucial for conceal endothelial cells to acquire the characteristic migratory phenotypes [28]. CDC42 activation is also required for EGF-induced cell migration and protrusion in carcinoma cells [29]. Therefore, in the present study, we aimed to interrogate whether the identified variant could result in the inactivation of CDC42 that is induced by either EGF or FGF medicated cell migration and proliferation. The results revealed that stimulation of normal fibroblast cells with either FGF or EGF treatments was associated with an increase in cell migration and cell proliferation when compared to the untreated cells control; however, the ability of either FGF or EGF to induce cell functions was abrogated when CDC42 is inactivated possibly due to the mutation that was identified in the patient cells. Our functional data are consistent with previous studies demonstrating that the dysfunction of CDC42 in patients with distinct disorders including immune deregulation, results in significant impaired cell migration and proliferation, indicating a dominant-negative effect [11,15,30]. The findings reported in this study extend the phenotypic spectrum resulting from CDC42 (p.P34Q) mutation and implies that patients with poor wound healing/recurrent infection can be screened for this germline mutation. The differences between the clinical features of the patient-reported here and those reported recently with similar mutations in the *CDC42* gene are of interest and warrant further investigations [11,15,21,30,31].

Previous studies have been reported that the mutated CDC42 protein can lead to impaired interaction with other effectors and regulator proteins, including WASP, RhoGDI, and IQGAP1, resulting in mislocalization, decreased cell migration, and aberrant cytoskeleton rearrangement [32,33,34]. Other studies showed that mutations in genes that functionally and structurally relevant to *CDC42,* like *RAC2*, as well as other genes, such as *DOCK8* [35], WAS [36], and *ARPC1B* [37], are associated with autoinflammation phenotypes and they cause aberrant migration and proliferation of myeloid and/or lymphoid cells. Therefore, it would be of interest to investigate whether the CDC42 (p.P34Q) mutation is involved in the regulation of these processes. 

Pro34 is a residue that is located at one of the loops (switch II) of CDC42 between the α1 and β2 domains that are observed near the nucleotide binding pocket and help in the stable binding of the RhoGDI with the CDC42 complex (Figure 3A). Pro34 to Gln34 substitution results in the establishment of new hydrophobic interaction between Val36 and Tyr51 and mutant Asp38 established ionic and hydrogen interaction with Lys50. These interactions might play a key role in CDC42 proper function and stability. This mutant Pro-to-Gln might destabilizes the switch II loop that is vital for signaling partners interactions might affect CDC42 trafficking and change in nearby surface electrostatic potentials. The different interaction patterns might affect the protein localization, as CDC42 has been observed in multiple cell compartments, such as plasma membrane, partly in the cytoplasm and different vesicles [38]. 

*CDC42* gene products are crucial for multiple systems in the body, including the immune system. CDC42 is involved in the actin cytoskeleton, vehicle trafficking, cell mobilities, regulation of transcription factors, proliferation, and reactive oxygen species production [39]. In mice, *CDC42* deletion resulted in lethal suppurative upper airway infections [40]. In another laboratory study, mice with the inducible deletion of CDC42 in B-cells resulted in reduced antibody responses, impaired B-cells homing to follicles in the spleen, and skewed cytokines response in CD4+ T-cells [41]. A Polish group described an 11-year-old boy with a de novo heterozygous mutation in *CDC42*. The patient had sinopulmonary infections, suppurative dermatitis, and severe varicella infections. The patient also had a severe inflammatory phenotype with autoinflammatory, HLH, and malignant lymphoproliferative disease. An immunological workup revealed pancytopenia and lymphopenia (mainly affecting T-helper cells and B-cells). Quantitive Immunoglobulins were also low [16]. Our patient had similar phenotypes. Both of the patients had recurrent suppurative sinopulmonary infections, pancytopenia, and lymphopenia. The lymphopenia was mainly a result of a decrease in the T-helper cells and B-cells. While the patient that was reported in the Polish study had low quantitive immunologlobulins, our patient had normal IgG, IgM, and IgA, as well as protective specific antibody responses to mumps, rubella, and tetanus.

The current study has multiple limitations, such as using samples from a single patient and performing indirect assays, to account for the link between CDC42 mutant and patients deficit. In addition, the clinical phenomena we reported, which could be caused by other independent or combined variants. Therefore, further investigations are required to confirm the pathogenicity of the candidate variant and its role in causing poor wound healing and immune response anomalies in the patient. 

## 5. Conclusions

The present work describes a novel de novo missense variant in the *CDC42* gene (c.101C > A:p.P34Q) in heterozygotic patients possibly contributing to poor wound healing that is associated with a large array of developmental processes, including pancytopenia, recurrent infections, and thrombocytopenia. Our results support previous findings and provide further explanation of the reported clinical phenotypes in association with dysfunction of the *CDC42* gene. These findings may provide further insight into the role of CDC42 in biological mechanisms that underlie cell migration anomalies. However, further investigations of the identified variant and other variants reported previously associated with migration and immune response anomalies in the animal model are needed in order to confirm these findings. 

## Figures and Tables

**Table 1:** Clinical description of our patient as competed to previously published cases of patients presenting with CDC42 mutations.

Clinical Phenotypes	Martinelli et al., 2018 [11]	Lam et al., 2019 [15]	Szczawinska-Poplonyk et al., 2020 [16]	Present Study	Total (%)
Intellectual disability	11/15	1/4	1/1	0/1	13/21 (61.9%)
Seizures	4/15	1/4	1/1	0/1	6/21 (28.5%)
Brain anomalies	9/15	1/4	1/1	1/1	12/21 (57%)
Optic atrophy	3/15	0/4	0/1	0/1	3/21 (14.3%)
Endocrine anomalies	4/15	0/4	0/1	0/1	4/21 (19%)
Facial dysmorphism	14/15	0/4	0/1	0/1	14/21 (66.7%)
Scoliosis/vertebral anomalies	5/15	0/4	0/1	0/1	5/21 (23.8%)
Camptodacyly or other digit anomalies	4/15	1/4	0/1	0/1	5/21 (23.8%)
Cardiac anomalies	7/15	0/4	0/1	0/1	7/21 (33.3%)
Recurrent infections	8/15	4/4	1/1	1/1	14/21 (66.7%)
Platelet anomalies (thrombocytopenia, macrothrombocytes	5/15	4/4	1/1	1/1	11/21 (52.4%)
Skin rash	0/15	4/4	1/1	1/1	6/21 (28.6%)
Hepatomegaly	0/15	4/4	1/1	0/1	5/21 (23.8%)
Splenomegaly	0/15	3/4	1/1	0/1	4/21 (19%)

## Data Availability

The datasets analyzed during the current study are available from the corresponding author on reasonable request.

## Supplementary Materials

The following are available online at https://www.mdpi.com/2073-4425/12/2/294/s1. Wound healing assay demonstrated that the mutated patient’s cells migrated very slowly com-pared to the normal cells. Pictures were taken for up to 24 h using EVOS FL Auto Imaging System at 10× magnification.
